# Nicotine Toxicity From Repeat Use of Nicotine Pouches

**DOI:** 10.1093/ntr/ntae111

**Published:** 2024-08-01

**Authors:** Jessica T Kent, Garrick Mok, Emily Austin

**Affiliations:** Division of Emergency Medicine, Department of Medicine, University of Toronto, Toronto, Ontario, Canada; Division of Emergency Medicine, Department of Medicine, University of Toronto, Toronto, Ontario, Canada; Department of Emergency Medicine, St. Michael’s Hospital, Toronto, Ontario, Canada; Division of Emergency Medicine, Department of Medicine, University of Toronto, Toronto, Ontario, Canada; Department of Emergency Medicine, St. Michael’s Hospital, Toronto, Ontario, Canada; Ontario Poison Centre, Toronto, Ontario, Canada

## Abstract

**Background:**

Nicotine pouches have emerged as a novel way to administer concentrated nicotine and come as a white powder in flavored, microfiber pouches placed between the cheek and gums to dissolve without requiring spitting. While marketed as a safe alternative to smoking, nicotine pouches have the potential for toxic exposure to users.

**Case Presentation:**

We present a case of a 21-year-old male with acute nicotine toxicity through repeated administration of nicotine pouches. Over the course of 12 hours, he consumed 15 extra-strength nicotine pouches (10.9 mg per pouch) as a study tool to prepare for the next-day exams. He presented to the emergency department with bizarre behavior requiring admission for persistent confusion and nausea which resolved after 24 hours.

**Conclusions:**

This case represents the first case of acute nicotine toxicity secondary to nicotine pouch use. These pouches are emerging as a novel way to use nicotine and present a serious risk of inadvertent overdose and harm.

**Implications:**

Nicotine pouches are emerging as a novel way to use nicotine, and second to e-cigarettes, are the most frequently used nicotine product among youth. These pouches, which lack clear warning labels, are promoted among social media forums and present a serious risk of inadvertent overdose and harm, especially among young adults. Healthcare professionals should be aware of this risk, especially from acute, repeated exposures, and should ensure the public is cautioned appropriately.

## Background

Nicotine is an alkaloid derived from tobacco leaves and is an addictive ingredient in tobacco products. Tobacco products are typically consumed by smoking cigarettes which do not deliver high concentrations of nicotine, thus limiting acute toxicity in adults. Modern products, like e-cigarettes, contain concentrated liquid nicotine and are implicated in an increasing number of toxic exposures.^1^ Nicotine pouches (NPs) have emerged as another novel way to administer concentrated nicotine as a flavored powder in microfiber pouches placed between the cheek and gums to dissolve without requiring spitting. Nicotine pouches are typically reported to contain between 2 and 10 mg of nicotine per pouch; however, have been reported to contain up to 47.5 mg per pouch in some countries.^2^ By comparison, a standard American combustible cigarette contains 0.56–1.53 mg per cigarette, delivering anywhere between 0.07 and 0.16 mg of nicotine per puff.^3^ Unlike cigarettes, NPs have a delayed time to peak (*T*_max_) but generally have greater nicotine maximum plasma concentrations (*C*_max_) and area under the plasma concentration–time curve (AUC) with doses as low as 4 mg nicotine per pouch.^4,5^ While marketed as a safe alternative to smoking, these pharmacokinetic properties of NPs have the potential to expose novice users to toxicity. We present a case of acute nicotine toxicity through repeated administration of NPs. To our knowledge, this is the first case of acute nicotine toxicity through nicotine pouch use identified in the literature.

## Case Report

A 21-year-old male presented to the emergency department by ambulance with depressed mental status after his brother noted him to be acting bizarrely: he was confused and responded nonsensically to questions. He was unable to sit in his chair and later slumped over the table, eventually sliding onto the floor. He was a nonsmoker; however, his brother reported the patient had used 15 extra-strength NPs (10.9 mg per pouch) over the preceding 12-hour period as a study aid, having read these pouches could promote alertness, as he had run out of his amphetamine/dextroamphetamine (last use 24 hours prior; one tablet, unknown dose). He initially presented nauseated, somnolent, and diaphoretic. His blood pressure was 184/99, heart rate 99, respiratory rate 17, oxygen saturation 98%, and temperature 36.3°C. His pupils were mydriatic (7 mm) and reactive bilaterally. He was tremulous; however, there was no focal weakness, muscle fasciculations, or clonus. Electrocardiogram and a noncontrast computed tomography scan of the head were unremarkable. His bloodwork demonstrated an elevated creatinine (118 umol/L; normal <93 μmol/L) for which he was treated with intravenous fluids but was otherwise unremarkable, including a serum drug screen which was negative for ethanol, acetaminophen, and salicylates. His depressed renal function was presumed to be secondary to dehydration. He was admitted for supportive treatment and continued to be nauseated, confused, and mildly agitated requiring fluids and benzodiazepines (a total of 2 mg lorazepam over 12 hours). Within 24 hours, he returned to baseline and was discharged home.

Qualitative urine testing by liquid chromatography with tandem mass spectrometry (LC-MS/MS) detected amphetamine, nicotine, and cotinine (nicotine metabolite), negative for all other substances.

## Discussion

This patient was exposed to 163.5 mg of nicotine over 12 hours which led to nausea, altered mental status, and hypertension. Typical features of moderate/severe nicotine toxicity include an early stage with headache, confusion, tremors, and restlessness, and a later stage with coma, convulsions, decreased reflexes, and weakness, which can progress to respiratory failure.^6^ Our patient presented with several clinical findings expected with moderate nicotine toxicity after a novel route of exposure, with repetitive subacute dosing of nicotine through NP. The reported ingestion 24 hours prior of a single tablet of amphetamine/dextroamphetamine was corroborated by urine analysis, but not believed to be a main contributor to clinical presentation because of the prominent features of confusion with a tremor, which are not seen with sympathomimetic toxicity. Pupillary mydriasis is relatively uncommon in acute nicotine poisoning and, therefore, a synergistic presentation of combined amphetamine and nicotine toxicity cannot be ruled out.

With the declining sales of nicotine cigarettes, tobacco companies have marketed novel nicotine delivery methods to increase uptake among a modern audience. Indeed, since their appearance on the US market in 2016, sales of NPs have increased dramatically with some e-commerce sites reporting 250% growth in most recent studies.^7^ These pouches are promoted among social media forums as study aids and safe alternatives to smoking and, second to e-cigarettes, are the most frequently used nicotine product among youth and young adults.^8–10^ The packages lack clear warning labels, and upon discharge, our patient admitted he had not realized could be harmful, reinforcing the serious risk for inadvertent overdose.

While marketed as a safe alternative to smoking, the pharmacokinetic (PK) properties of nicotine differ between NPs and e-cigarettes/combustible cigarettes. Volunteer PK studies suggest NPs (in doses as low as 4 mg of nicotine/pouch) result in a longer time to peak serum concentration (*T*_max_), and a larger peak concentration (*C*_max_) and AUC when compared to conventional American cigarettes (0.56–1.53 mg per cigarette). These imply a greater total systemic exposure and may put users at risk for acute toxicity after repetitive dosing.^4,5,10^ These properties would suggest that the user would experience a delay of the intended effects as well as a larger total nicotine exposure when compared to e-cigarettes to which youth would be more familiar.^3–5,10^ In summation, these properties may put users at risk of taking multiple doses to get the desired effect causing inadvertent toxicity as demonstrated in this case report, although further PK studies among real-world users are required.

The main limitation of this case study is the lack of quantitative LC-MS/MS testing available at our center. Without this testing, we were unable to perform a comparison of nicotine/cotinine levels to reference levels and relied on self-reporting.

## Conclusions

Nicotine pouches are emerging as a novel way to use nicotine and due to their unique pharmacokinetics, aggressive marketing, and lack of warning labels, present a serious risk for inadvertent overdose and harm, especially among young adults. Healthcare professionals should be aware of this risk, especially from repeated exposures on an acute basis, and should ensure the public is cautioned appropriately.
